# Supplementary material for: Sesamin Promotes Osteoporotic Fracture Healing by Activating Chondrogenesis and Angiogenesis Pathways
Source: Nutrients. 2022 May 18;14(10):2106. doi: 10.3390/nu14102106 (PMC9147588; doi:10.3390/nu14102106)
Supplement: Supplementary file 1 [file nutrients-14-02106-s001.zip › nutrients-1707639-supplementary.pdf]

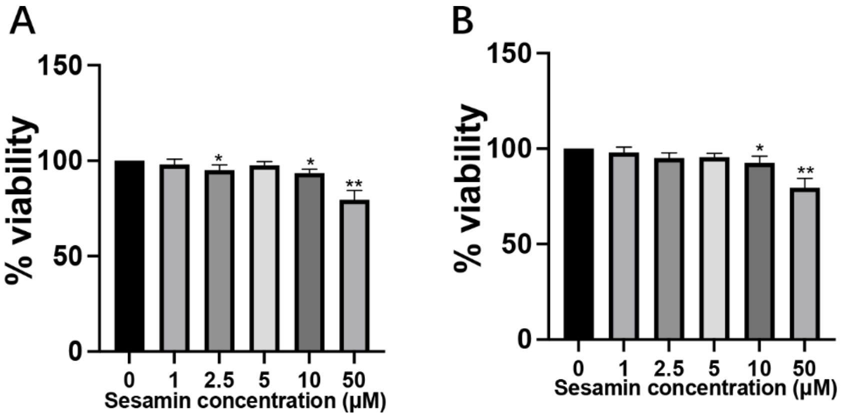

Figure S1. (**A**). Cytotoxicity assay of HUVECs by the treatment of sesamin at different concentration for 3 days ($n = 6$; * $p < 0.05$, ** $p < 0.01$). (**B**). Cytotoxicity assay of BMSCs by the treatment of sesamin at different concentration for 3 days ($n = 6$; * $p < 0.05$, ** $p < 0.01$).

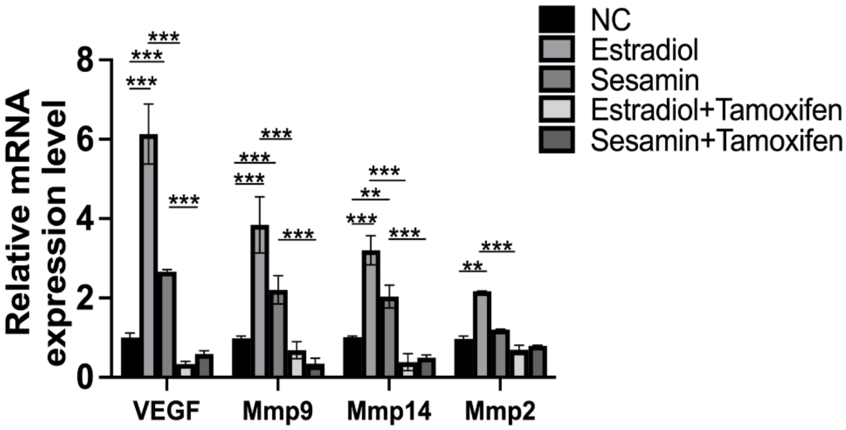

Figure S2. The mRNA expression level of angiogenesis markers in HUVECs treated with different combinations ($n = 6$; ** $p < 0.01$, *** $p < 0.001$).
